# Spinal autophagy is differently modulated in distinct mouse models of neuropathic pain

**DOI:** 10.1186/1744-8069-11-3

**Published:** 2015-02-02

**Authors:** Laura Berliocchi, Maria Maiarù, Giuseppe Pasquale Varano, Rossella Russo, Maria Tiziana Corasaniti, Giacinto Bagetta, Cristina Tassorelli

**Affiliations:** Department of Health Sciences, University “Magna Græcia” of Catanzaro, 88100 Catanzaro, Italy; Centre of Neuropharmacology of Normal and Pathological Synaptic Plasticity, University Consortium for Adaptive Disorders and Head Pain, 87036 Rende, Cosenza, Italy; Department of Pharmacy, Health and Nutritional Sciences, Section of Preclinical and Translational Pharmacology, University of Calabria, 87036 Rende, Cosenza, Italy; C. Mondino National Neurological Institute, Pavia, Italy; Department of Brain and Behavioral Sciences, University of Pavia, Pavia, Italy

**Keywords:** Spinal nerve ligation, Chronic constriction injury, Spared nerve injury, Neuropathic pain, Autophagy, LC3, SQSTM1/p62, Beclin 1, Chloroquine

## Abstract

**Background:**

Autophagy is a homeostatic degradative process essential for basal turnover of long-lived proteins and organelles as well as for removal of dysfunctional cellular components. Dysregulation of the autophagic machinery has been recently associated to several conditions including neurodegenerative diseases and cancer, but only very few studies have investigated its role in pain processing.

**Results:**

We previously described autophagy impairment at the spinal cord in the experimental model of neuropathic pain induced by spinal nerve ligation (SNL). In this study, we characterized the main autophagic markers in two other common experimental models of neuropathic pain, the chronic constriction injury (CCI) and the spared nerve injury (SNI). The different modulation of LC3-I, Beclin 1 and p62 suggested that autophagy is differentially affected in the spinal dorsal horn depending on the type of peripheral injury. Confocal analysis of p62 distribution in the spinal dorsal horn indicated its presence mainly in NeuN-positive cell bodies and occasionally in glial processes, thus suggesting a predominant expression in the neuronal compartment. Finally, we investigated the consequences of autophagy impairment on pain behaviour by using the autophagy blocker cloroquine. Intrathecal chloroquine injection in naïve mice induced spinal accumulation of LC3 and p62 paralleled by significant mechanical hypersensitivity thus confirming the block in autophagosome clearance and suggesting the participation of the autophagic process in spinal mechanisms of pain processing. Altogether, our data indicate that spinal autophagy is differentially altered in different experimental pain models of neuropathic pain and that this process may be relevant for pain control.

## Background

Autophagy is a degradative process by which cells dispose of cytosolic proteins and organelles [1]. It is an essential physiological mechanism for cellular differentiation, homeostasis and survival [2, 3] but it can also be induced by a series of environmental and cellular stresses such as nutrients starvation, trophic factors withdrawal and immune stimuli [4]. Autophagy is a multi-step, dynamic process initiated by the formation of autophagosomes. These are double membrane vesicles that, after engulfing cytosolic components and fusing with endosome (amphisomes step), will fuse with lysosomes and form autolysosomes for cargoes degradation [5]. Autophagy is also a highly regulated process and one of its key regulators is the kinase mammalian target of rapamycin mTOR, which in the presence of growth factors and abundant nutrients keeps autophagy down [6]. One of the key steps in autophagy regulation is the phosphatidylethanolamine conjugation to the microtubule-associated protein 1 light chain 3 (LC3-I) to form lipidated LC3-II, which binds to the expanding phagophore and remains associated with autophagosomes even after fusion with lysosomes [7].

Under normal conditions and particularly in neurons, autophagy remains at a low basal level and is essential for the maintenance of cellular homeostasis via the turnover of energy and cellular building material [8, 9]. However, in recent years, autophagy dysregulation has been implicated in an increasing number of pathological conditions such as metabolic diseases [10], neurodegenerative [11, 12] and inflammatory diseases [13], cancer [14] and glaucoma [15]. The mechanisms of autophagy dysfunction in these different pathological conditions are not clearly elucidated yet. For instance, although the importance of neuronal autophagy and its dysregulation in neurodegenerative disorders is undeniable, the exact events leading to autophagy malfunction, the key steps affected in each specific disease and their cause-effect relation remain unclear. Autophagosome accumulation has been observed in experimental models and patients in cases of neurodegenerative disorders associated with the misfolding and aggregation of specific mutated proteins such as Alzheimer’s, Parkinson’s and Huntington’s diseases [16–19]. Although it is still debated whether this would represent a protective or detrimental mechanism and a cause or a consequence of the disease, many studies indicate that autophagy operates as an efficient mechanism for the degradation of aggregation-prone proteins and pharmacological activation of autophagy has been suggested as a promising therapeutic strategy in many of these conditions [20].

In contrast to the great effort put into characterising the molecular mechanisms and the role of autophagy in chronic neurodegenerative disorders, so far only a few studies have investigated the role of autophagy in pain processing and whether dysregulation of this basic homeostatic process might be relevant also for pain conditions. Impairment of autophagy in the spinal cord after peripheral nerve injury was first described by our group following spinal nerve ligation (SNL) [21], and further confirmed by others in this same experimental model [22, 23]. More recently, a role for autophagy in Schwann cells during axonal peripheral nerve degeneration and pain *“chronification”* was also reported [24].

Here, we extended our initial study and investigated whether spinal modulation of the main autophagic markers LC3, Beclin 1 and p62 was common also to other widely used models of peripheral nerve injury such as the chronic constriction injury (CCI) of the sciatic nerve [25] and the spared nerve injury (SNI) model [26].

The different modulation of LC3, Beclin 1 and p62 suggests that autophagy is differentially affected in the spinal dorsal horn depending on the type of peripheral injury. To investigate autophagosomes distribution and cellular localization, p62 expression was investigated in the spinal dorsal horn and appeared strongly present in neurons. Finally, spinal block of autophagy with the autophagic flux inhibitor chloroquine induced increased mechanical sensitivity, thus suggesting that dysfunctional spinal autophagy may be relevant for altered nociceptive processing.

## Results

### Spinal nerve injuries induced a severe mechanical allodynia

**Figure 1:**
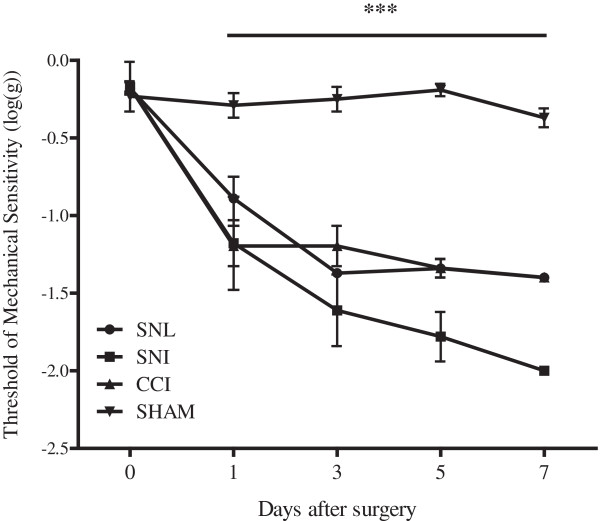
**Mechanical allodynia in different models of neuropathic pain.** A severe and persistent hypersensitivity developed following SNL, SNI and CCI surgery. Calibrated von Frey filaments were used to determine the withdrawal threshold for punctate mechanical stimulation. Data were expressed as means of ± SEM of threshold of mechanical sensitivity. ***P < 0.001 for all three models in comparison to the sham group. SNL n = 6, SNI n = 8, CCI n = 5.

In this study, three different models of neuropathic pain induced by peripheral nerve injury were used: the spinal nerve ligation (SNL) [27], the spared nerve injury (SNI) [26] and the chronic constriction injury (CCI) [25]. All these models induced a rapid reduction in threshold of mechanical sensitivity on the injured side compared to the sham group (Figure 1), but not on the contralateral side, as previously described [25–27]. In the SNL model, the threshold of mechanical sensitivity dramatically decreased 1 day after surgery (Figure 1) and remained constant for at least 28 days, as previously described [21]. In the SNI model, a reduction in threshold was observed starting 1 day after surgery; maximal mechanical sensitivity was reached at 7 days (Figure 1) and kept constant for at least 14 days (data not shown) [26]. After CCI of the sciatic nerve, a robust mechanical allodynia developed starting from 1 day after surgery (Figure 1) and lasting for at least 28 days as previously described [28]. In all three models, the difference in mechanical sensitivity between the injured group and the respective sham group was statistically significant (p < 0.001, determined by two-way ANOVA).

### Autophagy modulation following spinal nerve injury

Microtubule-associated protein 1 light chain 3 (LC3) was the first mammalian protein discovered to be specifically associated with autophagosomal membranes [7]. It exists in a non-lipidated and a lipidated form, usually referred to as LC3-I and LC3-II, respectively. Because of its essential role in the expansion step of autophagosome formation, LC3-II is regarded as the most reliable marker protein for macroautophagy [29].

**Figure 2:**
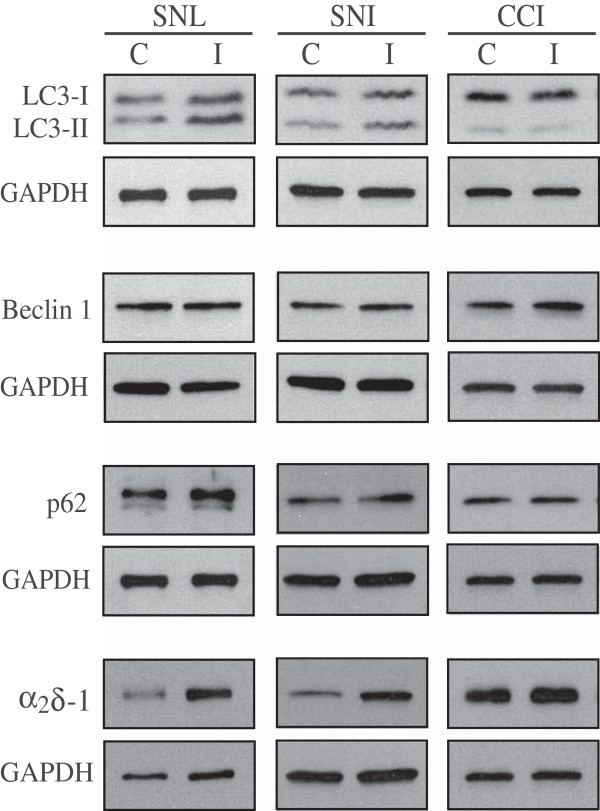
**Expression of autophagic markers in the spinal dorsal horn in three different models of peripheral nerve injury (SNL, SNI and CCI).** The representative Western blots show levels of the autophagic markers LC3-I, LC3-II, Beclin 1 and p62 and voltage-gated calcium channel subunit α_2_δ-1 in the spinal dorsal horn ipsilateral (I) and contralateral (C) to the side of injury.

**Figure 3:**
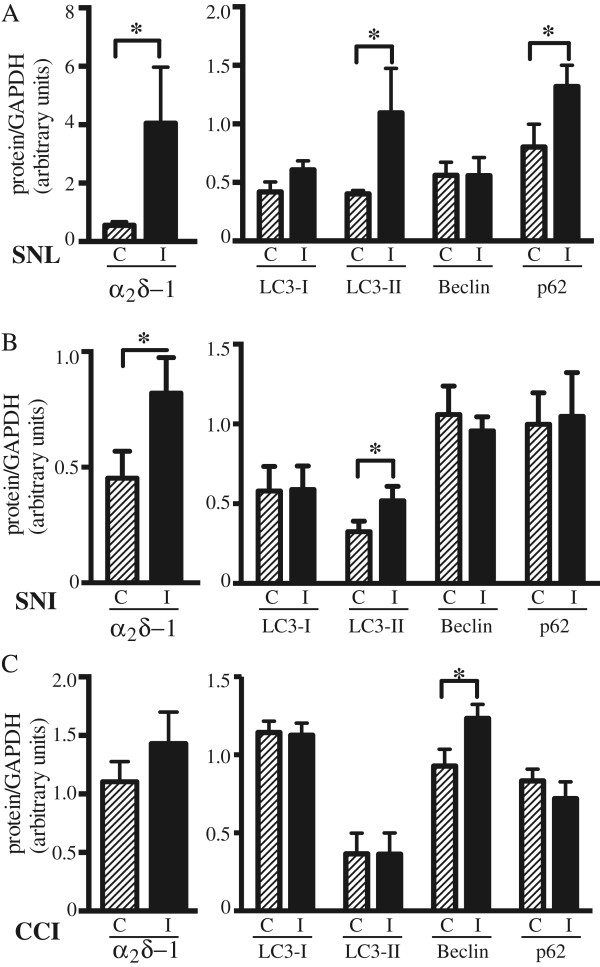
**Densitometric analysis of autophagic markers changes detected by Western blot in the spinal dorsal horn following three different models of peripheral nerve injury (SNL, SNI and CCI).** The SNL model **(A)** was characterised by increased LC3-II and p62, whereas in the SNI **(B)** and CCI **(C)** only LC3-II and Beclin 1 increase were detected, respectively. A statistically significant upregulation of the voltage-gated calcium channel subunit α_2_δ-1 was observed in the injured side of the SNL and the SNI model. Signals from each band were normalised towards the corresponding GAPDH signal. Values were expressed as mean ± SEM. *P < 0.05, SNL n = 8, SNI n = 5, CCI n = 5.

The expression of LC3 was examined by Western blot analysis in the spinal dorsal horn 7 days after SNL and CCI, and 14 days following SNI (Figure 2). Increased levels of LC3-II in the ipsilateral (I) versus the contralateral (C) dorsal horn were observed in the SNL model as shown by Western blot (Figure 2) and by densitometric and statistical analysis (p < 0.05; Figure 3). Similarly, a statistically significant increase in LC3-II levels was observed in the ipsilateral dorsal horn after SNI but not after CCI (Figures 2 and 3B and C). No statistically significant variation in LC3-I expression was detected in any of the three models (Figures 2 and 3).

Autophagic activity does not only rely on increased synthesis or lipidation of LC3, but on a series of regulatory proteins orchestrating different autophagic steps from formation of autophagosomes to fusion with the lysosomes and subsequent release of breakdown products. Here, we first analysed the expression of Beclin 1, a key protein in the induction of autophagy essential for autophagosomes formation [30]. To understand whether LC3-II formation observed in the SNL and SNI models was due to an upstream induction of autophagy, the expression of Beclin 1 was analysed. A statistically significant increase of Beclin 1 was observed on the injured side of the spinal dorsal cord in the CCI model (p < 0.05; Figures 2 and 3C). On the contrary, no significant difference in Beclin 1 expression was observed after SNI (Figures 2 and 3B), as well as SNL (Figures 2 and 3A), thus suggesting a different upstream modulation of autophagy in the three models.

Besides LC3, levels of autophagy substrates can be used to monitor autophagic flux. Among these, p62/SQSTM1 is one of the best-known and more widely used [1]. p62 is selectively incorporated into autophagosomes through direct binding to LC3 and efficiently degraded in functional autolysosomes [31, 32], thus serving as a readout of autophagic degradation. Therefore, p62 analysis may provide useful information on the final degradative steps of autophagy. We have previously shown that in the SNL model, LC3-II formation is accompanied by p62 accumulation suggesting that increased LC3-II levels might be a consequence of defective autophagosome clearance rather than autophagy induction [21]. To verify this hypothesis also in the case of injury induced by SNI or CCI, p62 levels were evaluated by Western blot in the spinal dorsal horn of mice that underwent the three different types of surgery.

While SNL induced a statistically significant accumulation of p62 on the side ipsilateral to injury (Figures 2 and 3A), no differences in p62 levels were observed between the contra- and ipsi-lateral side following SNI or CCI of the sciatic nerve (Figures 2 and 3B and C).

As in our previous work [21], we used the calcium channel subunit α_2_δ-1 as biochemical marker of a neuropathic pain state because of its known upregulation ipsilaterally to the injury and its confined expression to the correspondent lumbar portion of spinal cord following SNL [33]. A statistically significant upregulation of the α_2_δ-1 subunit was detected in the dorsal spinal cord of the injured side in SNL and SNI groups, although not after CCI (Figures 2 and 3) and was used in all experiments as internal control for correct cord sampling. The lack of a significant upregulation in the CCI model is in line with the previous observation that the upregulation of this subunit is correlated to the severity of the injury [34]. The densitometric analysis of the calcium subunit and the different autophagic markers is plotted in Figure 3 according to the model.

### p62 is mainly expressed in neurons in the spinal dorsal horn following spinal nerve ligation

**Figure 4:**
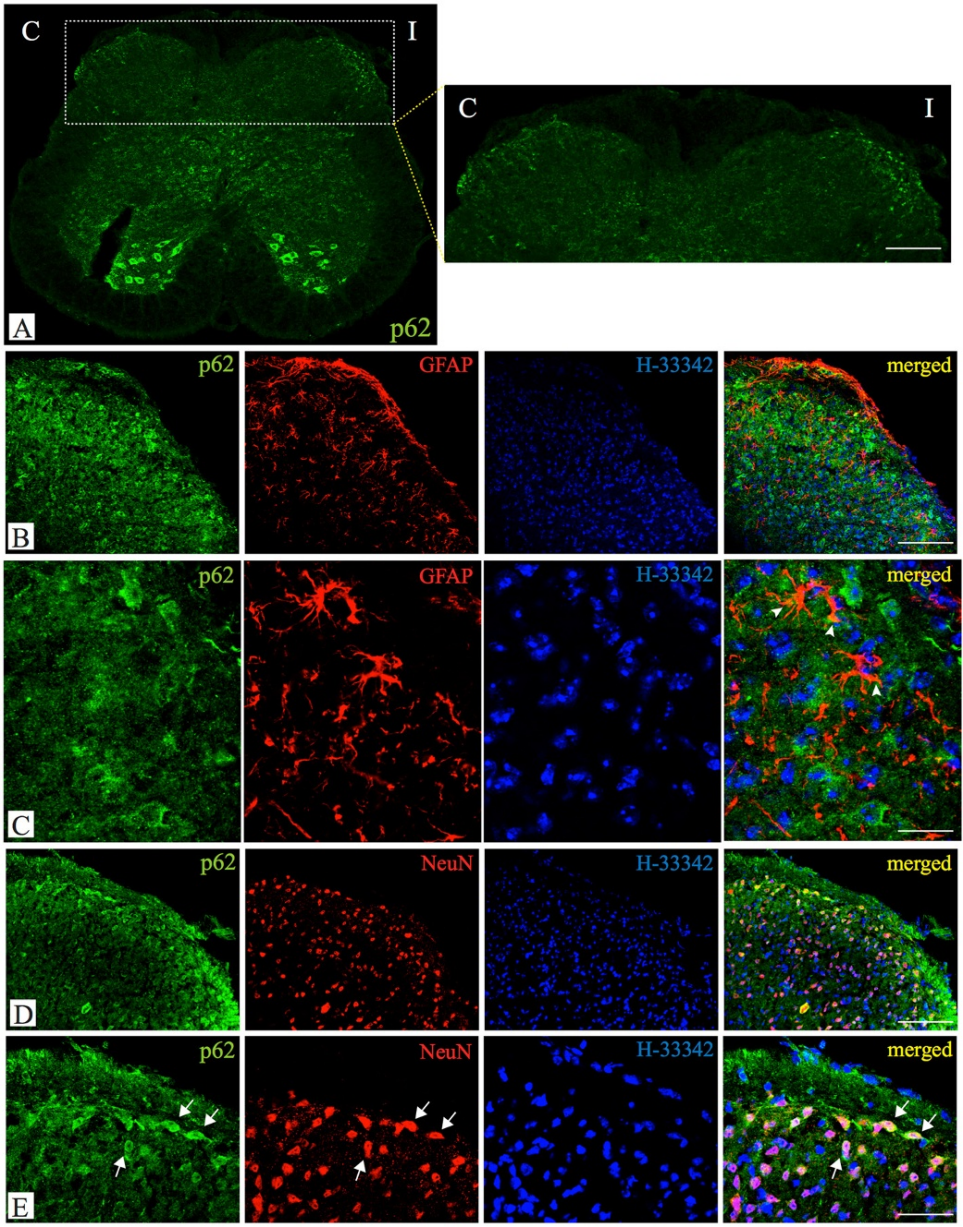
**Distribution and cellular localisation of p62 immunoreactivity in the spinal dorsal horn following SNL. (A)** Although ubiquitously expressed (see positive cell bodies in the ventral spinal cord), high levels of p62 were present in the superficial layers of the dorsal horn, particularly on the side ipsilateral to injury **(A, insert)**. Spots of p62 immunoreactivity were detected within glial processes as shown by some p62 immunolabelling within GFAP-positive structures (**B** and **C** arrowheads). However, high p62 levels were particularly evident in some neuronal cell, as shown by the strong signal within NeuN-positive cell bodies (**D** and **E** arrows). Each image is a single focal plane from spinal cord sections of mice undergone SNL; B, C, D, E show ipsilateral side. Scale bars: A 250 μm; B, D 100 μm; C 20 μm; E, 50 μm.

Currently available antibodies are not able to distinguish between cytosolic LC3-I and autophagosome-bound LC3-II. We selected the autophagic substrate p62 to investigate autophagosomes localization and distribution in spinal dorsal horn following peripheral nerve injury induced by SNL. Immunohistochemistry showed that p62 was ubiquitously expressed in the spinal cord, with high levels of expression in the superficial layers of the ipsilateral (I) dorsal horn following SNL (Figure 4). To further characterize the cellular localization of p62, double immunofluorescent labellings were performed using antibodies against p62 combined with the neuronal marker NeuN, the astrocytic marker GFAP or the microglial marker Iba-1 (not shown). Imunolabelling showed a main presence of p62 in NeuN-positive cell bodies (Figure 4) but spots of p62 immunoreactivity could also be detected within glial processes, as shown by the presence in GFAP-positive structures (Figure 4). A certain degree of p62 immunoreactivity was present in the neuropile suggesting that p62 might be expressed also in neuronal processes.

### Spinal block of autophagy results in pain behaviour

**Figure 5:**
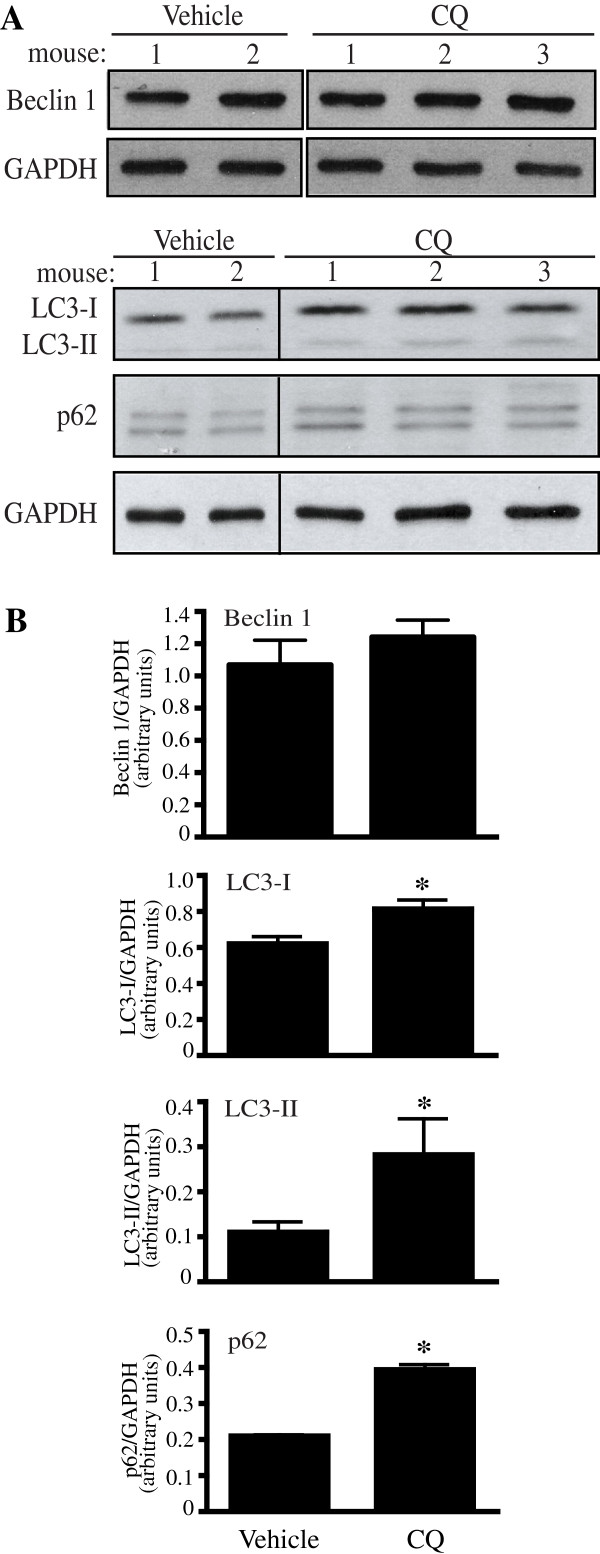
**Effect of chloroquine intratechal injection on Beclin 1, LC3 and p62 in the spinal dorsal horn. (A)** Representative Western blots of Beclin 1, LC3 and p62 protein levels after chloroquine (300pmoles/3 μl, i.t.) according to the treatment protocol in Figure 6A. **(B)** Densitometric analysis of Beclin 1, LC3-II and p62 expression. While Beclin 1 expression was not affected, LC3-I, LC3-II and p62 levels were significantly increased in the chloroquine group compared to the vehicle-injected group. The appearance of a second band has been previously suggested as a p62 splicing variant or a partially cleaved product [42]. Signals of each band were normalised to the respective GAPDH. Data were expressed as mean ± SEM. *P < 0.05. Vehicle n = 3, choloroquine (CQ) n = 6 (n = 5 for Beclin 1).

**Figure 6:**
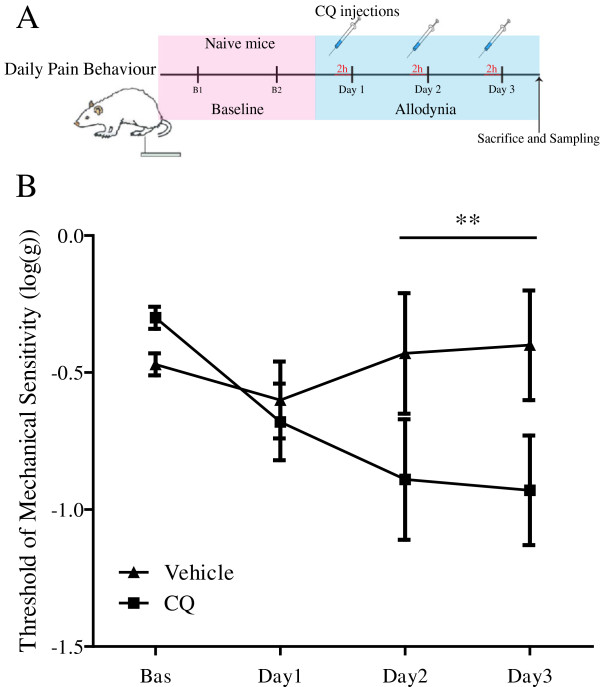
**Effect of chloroquine intratechal injection on mechanical sensitivity. (A)** Baseline mechanical thresholds were assessed in naïve mice for 2 consecutive days before the first injection. Chloroquine (300pmoles/3 μl) or vehicle were then injected intrathecally once daily. Two hours after treatment, mechanical sensitivity was measured by Von Frey’s test. The protocol was repeated for three consecutive days. **(B)** Localised spinal block of autophagy significantly reduced the threshold of mechanical sensitivity, in comparison to vehicle-treated mice. Data were expressed as mean ± SEM of the threshold of mechanical sensitivity. **P < 0.01. Vehicle n = 3, chloroquine (CQ) n = 6.

In order to verify the relevance of a downstream impairment of autophagy at spinal cord level and its consequences for pain processing, the anti-malaria drug chloroquine was used as a pharmacological tool to block autophagy. Together with bafilomycin A1 and NH_4_Cl, chloroquine is one of the main chemicals used to inhibit autophagy [29]. Like NH_4_Cl, chloroquine neutralises the lysosomal pH and by inhibiting endogenous protein degradation causes the accumulation of sequestrated material in either autophagosomes or autolysosomes. Moreover, chloroquine has been suggested to block autophagosomes fusion with lysosomes [29]. Chloroquine was administered to naïve mice by a daily intrathecal injection at the dose of 300pmoles/3 μl for 3 consecutive days. The behavioural test was conducted daily 2 h after injection for 3 days. On the third day, mice were sacrificed after the test and the spinal dorsal horn dissected for Western blot analysis (Figure 5). Chloroquine treatment induced LC3-II formation and p62 accumulation in treated mice, when compared to vehicle-injected mice, but did not affect Beclin 1 levels (Figure 5) thus confirming the drug action on a downstream step of the autophagic process. Moreover, the behavioural test (von Frey’s) carried parallel to the treatment (Figure 6A) showed a significant reduction in threshold of mechanical sensitivity starting from day 2 in chloroquine-injected mice in comparison to vehicle-injected mice (p < 0.01 determined by two-way ANOVA; Figure 6B) thus suggesting that impaired spinal autophagy can result in altered mechanical sensitivity.

## Discussion

Pain is a common feature of many neurodegenerative diseases [35–37], in which autophagy is being extensively studied as an important player in the progression of the pathology [17], and indicated as a new promising therapeutic target [37]. Understanding the role of autophagy in the mechanisms of pain processing may represent a valuable tool in pain management also in these specific patients populations.

So far only a few studies have investigated the role of autophagy in pain processing. Impairment of autophagy in the spinal cord following peripheral nerve injury was first described in the spinal nerve ligation (SNL) model by our group [21] and further confirmed in this same experimental model by others [22, 23]. Here, we investigated whether spinal modulation of some of the main autophagic markers (i.e. LC3, Beclin 1 and p62) observed following SNL was common also to other widely used models of peripheral nerve injury such as the chronic constriction injury (CCI) of the sciatic nerve [25] and the spared nerve injury (SNI) model [26]. In the SNL, increased LC3-II levels were paralleled by strong p62 accumulation but no significant LC3-I and Beclin 1 increase, suggesting the occurrence of a block in the late phases of the autophagic flux [21] rather than an induction of the process.

Indeed, LC3-II upregulation may be indicative of increased autopagic flux but also of defective autophagosome clearance. In this last case, LC3-II upregulation will be associated to p62 accumulation, as this autophagy substrate will not be efficiently degraded by the autophagosomes [29, 38].

The SNI and the CCI models showed completely different patterns, with no significant change in LC3-I expression in any of the two models (Figures 2 and 3) and LC3-II, the lipidated LC3 form known to be associated with autophagosomes [7], being significantly increased in the dorsal spinal cord ipsilateral to injury following SNI but not CCI (Figures 2 and 3). Monitoring LC3-II conversion is considered one of the most reliable methods for monitoring autophagy. However, a concomitant increase in both the rate of autophagosome formation and LC-3 downstream degradation can show normal steady-state levels in LC3-II, despite enhanced autophagy activity [39]. Moreover, LC3 accumulation can result from autophagy induction, but also from its impairment at one of the last steps such as fusion with the lysosomes or cargo degradation [38]. Therefore, it is preferable to integrate LC3 studies with the analysis of other components of the autophagic machinery such as members of the initiation complex (i.e. Beclin 1) or autolysosomes substrates (i.e. SQSTM1/p62) [29]. Studies on the regulatory role of Beclin 1 on autophagy suggested that Beclin 1 complex is involved in autophagosome formation at an early stage [30] and this complex is essential for the recruitment of other Autophagy-related (Atg) proteins to the pre-autophagosomal structure [40]. In the SNL model, LC3-II was not associated to any significant ipsilateral increase of LC3-I and Beclin 1 [21] and, similarly, no differences in LC3 and Beclin 1 were detected between the ipsi- and contralateral side of dorsal spinal cord following SNI (Figures 2 and 3). On the contrary, Beclin 1 was clearly upregulated in the ipsilateral side compared to the contralateral following CCI, suggesting a possible induction of autophagy in this model.

One of the best-known autophagic substrates is p62/SQSTM1, a key LC-3-binding protein, which serves as a link between LC-3 and ubiquitinated proteins [32]. p62 and p62-bound polyubiquitinated proteins become incorporated into the completed autophagosome and are degraded in autolysosomes. Because of the correlation between autophagy modulation and p62 levels [32, 41, 42], this substrate is considered a useful readout of autophagic degradation [29, 43]. Indeed, p62 levels increase when autophagy is impaired [42]. In the SNL model, ipsilateral p62 accumulation was observed in the presence of high LC3-II levels without changes in LC3-I and Beclin 1 expression, thus pointing towards a block in the final degradative steps of autophagy [21]. On the contrary, no differences in p62 levels were observed between the ipsi- and contra-lateral side following CCI and SNI (Figures 2 and 3). Because of the spinal accumulation detected following SNL by Western blot analysis, the distribution and cellular localisation of p62 in the spinal dorsal horn was also investigated by confocal microscopy in this model. The protein appeared ubiquitously expressed in the spinal cord, with high levels of expression also in cell bodies of the ventral spinal cord, most probably motorneurons. In the superficial layers of the dorsal horn, p62 immunolabeling appeared increased in the side ipsilateral to injury. It was mainly expressed in NeuN-positive cell bodies, but spots of p62 immunoreactivity could be detected in glial processes as shown by p62 imunolabelling within GFAP-positive structures. Immunoreactivity was also observed in the neuropile suggesting that p62 might be expressed also in neurites. Further morphological and functional studies will be needed to verify the presence and the role of the autophagic machinery in this specific cellular compartment that could be a relevant component in pain signalling. Indeed, accumulating evidence has revealed that, not only is autophagy uniquely regulated in neurons, but it may also have a distinct function and regulation within the axonal compartment, the dendrites and the soma [44, 45]. Autophagy participation in axon homeostasis has been recently suggested by degeneration of axon terminals induced in non-damaged neurons by autophagy suppression [46]. Also, a close relationship between the autophagic process and neuritic alterations has been suggested in several *in vitro* and *in vivo* models of neurites dystrophies [47]. Local autophagy can sequester presynaptic components and therefore modulate presynaptic function [48] as well as selectively degrade postsynaptic receptors [49, 50]. In addition to the well-established role in cellular homeostasis and stress response, the emerging role of autophagy in neurotransmitter release modulation and synaptic as well dendritic remodelling may reveal important also in the context of the structural and functional changes occurring at spinal dorsal horn during chronic pain states [51, 52]. Although LC-3 and Beclin 1 co-localisation with calretinin-positive neurons in the spinal cord has been recently reported [22], further functional studies are mandatory to verify whether autophagy dysregulation may play a role in the loss of GABA inhibitory tone [53, 54] or in the glutamate excitatory unbalance [55] observed in the spinal cord following peripheral nerve injury. Also, the observation that morphine can induce Beclin 1 and Atg5-dependent autophagy in human neuroblastoma SH-SY5Y cells and in the rat hippocampus [56] together with the recent report that autophagy in the spinal dorsal horn might play a role in morphine tolerance [57] seem to support an involvement of this process in the mechanisms of central sensitization occurring at the spinal cord. Altogether, the analysis of LC3, Beclin 1 and p62 indicated the occurrence of distinct patterns of autophagy modulation in the three models studied. While a block in completion of basal autophagy seemed to be associated with SNL injury, spinal autophagy appeared increased and functional following CCI of the sciatic nerve. The trend of spinal autophagy modulation after SNI appeared comparable to what observed following SNL, though not all changes were statistically significant. Although all three models of peripheral nerve injury produce signs of both ongoing and evoked pain and similar behavioural phenotypes, differences in the nature of injury, in the extent of neuronal damage and lesion site [58] may be responsible for the different molecular alterations observed in respect to autophagy modulation in the spinal dorsal horn. Because of p62 accumulation observed following SNL, we hypothesised that in this model a block may have occurred at the final steps of autophagy degradation. A transitory beneficial effect of autophagy inhibition on pain behaviour has been recently shown in the SNL model [22]. However, autophagy was inhibited with 3-methyladenine (3-MA), a phosphatidylinositol 3-kinase (PtdIns3K) inhibitor that effectively blocks an early stage of autophagy by inhibiting the class III PtdIns3K but that can also promote autophagy by inhibition of the class I enzyme [29, 59]. Here, we verified the relevance of spinal autophagy for pain processing by blocking the autophagic flux locally at the lumbar spinal cord by intrathecal injection of the autophagy blocker chloroquine. Chloroquine is a lysosomotropic agent that elevates the lysosomal pH inactivating lysosomial enzymes but also the fusion step between autophagosomes and lysosomes. By doing so, it induces a block of autophagic flux and inhibits the degradation of the autophagic cargo thereby blocking the late stage of autophagy [29]. When injected intrathecally in naïve mice, chloroquine was able to modulate the spinal autophagic machinery as shown by the increase in LC3-II and p62, indicative of autophagosomes accumulation (Figure 5). Moreover, in these same animals, chloroquine induced a significant reduction in threshold of mechanical sensitivity (Figure 6) supporting the concept that impaired autophagy may contribute to pain.

The exact mechanisms through which autophagy may be linked to mechanisms of abnormal spinal plasticity in pain signaling need to be further elucidated. However, several drugs are able to modulate autophagy at different steps either as inhibitors or activators [60] and a number of clinical trials [61, 62] are currently evaluating their use in human conditions, such as neurodegenerative diseases and cancer, often associated with pain syndromes. Therefore, a better understanding of the role of this physiological process also in the context of pain appears to be relevant for a better management of chronic conditions and as a potential novel tool for pain control.

## Material and methods

### Animals

Male C57Bl/6 mice (20-22 g) (Charles River, Italy) were used for all experiments. Animals were kept in their home cages at 21°C and 55% relative humidity with a light–dark cycle of 12 am (lights on at 08:00 am); food and water were provided *ad libitum*. Experimental protocols were in accordance to the guidelines of the Italian Ministry of Health for animal care (D.L. 116/1992). All efforts were made to minimise animal suffering and to reduce the number of animal used.

### Surgery

Spinal Nerve Ligation

The spinal nerve ligation (SNL) was performed according to the Kim & Chung model [27]. Under 2% isoflurane anaesthesia, a middle incision was made in the skin of the back at the L2-S2 levels and the left paraspinal muscles separated from the spinal process at the L4-S1 levels. The left lumbar L6 transverse process was carefully removed to identify the L4 and L5 spinal nerves. The left L5 spinal nerve was isolated and tightly ligated with a 6–0 silk thread. Complete hemostasis was confirmed and the wound was sutured. The surgical procedure for the sham group was identical to the SNL group, except that the spinal nerve was not ligated.

Spared Nerve Injury

The spared nerve injury (SNI) was performed as described by Decosterd and Woolf [26]. Under 2% isoflurane anaesthesia the skin on the lateral surface of the thigh was incised and a section made directly though the biceps femoris muscle exposing the sciatic nerve and its three terminal branches: the sural, common peroneal and tibial nerves. The common peroneal and the tibial nerves were tight-ligated with 5–0 silk and sectioned distal to the ligation. Great care was taken to avoid any contact with or stretching of the spared sural nerve. Complete hemostasis was confirmed and the wound was sutured. For sham control, the procedure involved exposure of the sciatic nerve and its branches without any lesion.

Chronic Constriction Injury

Chronic constriction injury (CCI) of the sciatic nerve was performed according to the model described by Bennet & Xie [25]. Under 2% isoflurane anaesthesia, the common sciatic nerve was exposed and dissected from the surrounding connective tissue. Three loosely constrictive ligatures (5–0 silk) were tied around the nerve with a 1–1.5 mm distance between ligatures. The muscle and skin were sutured after complete hemostasis was confirmed. For sham surgery, the sciatic nerve was exposed as described above but no contact was made with the nerve.

For all the models, after surgery foot posture and general mice behaviour were monitored throughout the postoperative period.

### Drug treatment

Chloroquine (Sigma, c6628) was dissolved in saline to have a final 100 μM solution. Mice were divided into two groups, one treated with chloroquine and the other one treated with vehicle (saline). Intrathecal injection (i.t.) was performed as previously described [63, 64]. Briefly, the i.t. injections were given by percutaneous lumbar puncture through an intervertebral space at the level of the 5th or 6th vertebrae. The drugs were administered i.t. in a volume of 3 μl using a 10 μl Hamilton microsyringe. A flick of the tail was used as an indication that the needle had penetrated the *dura mater*. After injection the syringe was rotated and removed and posture and locomotion were checked. A 3 μl i.t. injection was performed once a day for three days. After injection animals were kept under observation and after 2 h the behavioural test was performed.

### Behavioural test

Animals were placed in Plexiglas chambers, located on an elevated wire grid and allowed to habituate for at least 1 hour. After this time, the plantar surface of the paw was stimulated with a series of von Frey monofilaments of ascending forces [27, 65, 66]. The threshold was determined by using the up-down method as described by Chaplan and colleagues [67] and the data were expressed as log of mean of the pain threshold ± SEM.

### Immunohistochemical analysis

Animals were deeply anaesthetized with pentobarbital and perfused transcardially with saline containing heparin followed by 4% paraformaldehyde (PFA) in 0,1 M phosphate buffer (PB). The lumbar spinal cord was dissected out, post-fixed in 4% PFA for 2 h and then transferred into a 30% sucrose solution in PB containing 0,01% azide at 4°C, for at least 24 h. The spinal cord was sectioned on cryostat set at 20 μm thickness. Sections were incubated with a primary antibody against p62 (anti-p62/SQSTM1, PM045, MBL), followed by incubation with the appropriate Alexa Fluor 488-conjugated secondary antibody. For double immunostainings, sections were sequentially incubated also with anti-GFAP (astrocyte marker; MAB360, Millipore) or anti-NeuN (neuronal marker; MAB377, Millipore) or anti-Iba1 (microglial marker; 019–19741, Wako) antibody followed by incubation with the appropriate Alexa Fluor 594-conjugated secondary antibody. Nuclei were counterstained with H-33342 (Life Technologies) and all sections cover slipped with Fluoromount Aqueous Mounting Medium (Sigma). Controls were included omitting the first or second primary antibodies. Images were acquired by laser scanning microscopy (Leica TC-SP2 Confocal System; Leica Microsystems, Milan, Italy) using sequential scanning and 10X (HC PL Fluotar10X/0.30), 40X (HCX PL APO 40X/1.25) or 63X (HCX PL APO63x/1.4) objectives (Leica Microsystems, Milan, Italy).

### Western blot analysis

For Western blot analysis, animals were terminally anaesthetized with CO_2_ and the spinal cord segment corresponding to the lumbar area was rapidly removed. The ipsi- and contra-lateral dorsal horn quadrants L4-L5 were quickly dissected out and frozen in liquid nitrogen, then stored at −80°C until further processing. Each sample was homogenised in ice-cold lysis buffer (50 mM Tris–HCl, pH 8, 150 mM NaCl, 1 mM EDTA, 0,1% SDS, 1% IGEPAL, 0,5% Na-deoxyxolate) in the presence of protease inhibitors (Sigma, P8349) and incubated on ice for 40 min. Samples were then centrifuged at 14,000 x g for 15 min at 4°C. Total protein content was determined in the supernatants by the Bio-Rad DC Protein Assay Kit (Bio-Rad Laboratories, Milan, Italy). Equal amounts of total proteins were separated by sodium dodecyl-sulfate polyacrylamide gel electrophoresis (SDS-PAGE, 15%) and transferred onto PVDF membranes (Immobilon-P, Sigma). After blocking for 1 hour at room temperature in Tris-buffer saline containing 0,05% Tween 20 (TBST) and 5% non-fat milk, the membranes were incubate with primary antibody: anti-LC3 (1:2000, MBL); anti-Beclin (1:4000, MBL); anti-p62-SQSTM1 (1:1000, MBL); anti-α_2_δ-1 (1:1000, Sigma-Aldrich); anti-GAPDH (1:40000, Ambion). After several washes, an appropriate HRP-conjugated secondary antibody (goat IgG; Pierce Bio-tecnology, USA) was applied for 1 hour at room temperature. Peroxidase activity was visualized using the ECL Western Blotting Detection kit (ECL, Amersham Biosciences, Italy) and X-ray films (Hyperfilm ECL, Amersham Bioscence). Signal intensity was measured using Fiji software (NIH, USA). For quantitative analysis, the Beclin 1, LC3-I, LC3-II, p62 and α_2_δ-1 signals of each sample were normalized towards the corresponding GAPDH signal.

### Statistic

Data were expressed as mean ± SEM and statistically assessed for differences either by two-way ANOVA (behavioural tests) or by Student’s t-test. Calculations were done using GraphPad Prism (GraphPad Software Inc or SPSS software, Chicago, IL).

## Authors’ informations

Current affiliations: MM, Department of Cell and Developmental Biology, University College London, London WC1E 6BT, UK.

GV, Department of Pharmacy, Health and Nutritional Sciences, Section of Preclinical and Translational Pharmacology, University of Calabria, 87036 Rende (Cosenza), Italy.

## Abbreviations

Atg: Autophagy-related proteins
mTOR: Mammalian target of rapamycin
LC3: Microtubule-associated protein 1 light chain
SNL: Spinal nerve ligation
CCI: Chronic constriction injury
SNI: Spared nerve injury
SQSTM1: Sequestosome-1
GFAP: Glial fibrillary acidic protein
CQ: Chloroquine
PtdIns3K: Phosphatidylinositol 3-kinase.
